# Assessing Complexity in Physiological Systems Through Biomedical Signals Analysis II

**DOI:** 10.3390/e27121185

**Published:** 2025-11-21

**Authors:** Paolo Castiglioni, Luca Faes, Gaetano Valenza, Andrea Faini

**Affiliations:** 1 Department of Biotechnology and Life Sciences (DBSV), University of Insubria, 21100 Varese, Italy; 2 IRCCS Fondazione don Carlo Gnocchi, 20148 Milan, Italy; 3 Department of Engineering, University of Palermo, 90128 Palermo, Italy; luca.faes@unipa.it; 4 Department of Information Engineering and Research Center “E. Piaggio”, University of Pisa, 56122 Pisa, Italy; g.valenza@ing.unipi.it; 5 Department of Cardiovascular, Neural and Metabolic Sciences, Istituto Auxologico Italiano, IRCCS, 20149 Milan, Italy; a.faini@auxologico.it; 6 Department of Electronics Information and Bioengineering, Politecnico di Milano, 20156 Milan, Italy

Physical systems are widely characterized in terms of their complex dynamics in physiology and medicine to understand the ability of a living system to adapt to external perturbations. Complex dynamics are typical of systems characterized by physical structures with fractal geometry and nonlinear components, often composed of numerous interdependent elements that self-organize, interacting at different hierarchical levels and time scales. This allows homeostasis to be maintained even in the presence of external perturbations and interactions with other physiological systems, through neural and humoral networks.

The variables that fully describe the states of physiological systems often remain inaccessible, but it is possible to observe the temporal evolution of related biosignals. Their analysis may theoretically allow for the extraction of information about the complex dynamics of the system under study. Typical measures are related to the unpredictability and self-similarity of the recorded time series, as well as the amount of information exchanged between biosignals. These complexity measures are proving increasingly valuable for better understanding the functioning of a physiological system, monitoring its health over time, or predicting pathological events.

Therefore, the lively interest in the complexity analysis of biomedical signals has prompted us to propose this Special Issue, “Assessing Complexity in Physiological Systems through Biomedical Signals Analysis II”, as a continuation of the Special Issue on complexity that we published in this same journal a few years ago [1]. The present Special Issue brings together thirteen contributions that address, from different methodological and applicative perspectives, the topic of complexity in physiological systems. The systems studied in this issue are the cardiovascular and central nervous systems. However, a clear separation between systems is impossible because psychological factors, cognitive loads, and the heart–brain connection via the autonomic nervous system, for example, produce important interactions between these systems, as well as with other regulatory systems in the body, such as those responsible for motor and respiratory control.

A total of five contributions concern heart rate variability (HRV). The paper “Heart Rate Complexity and Autonomic Modulation Are Associated with Psychological Response Inhibition in Healthy Subjects” [2] studied HRV indices linked to entropy and sympathovagal balance in healthy volunteers during the execution of cognitive tests to assess flexibility, inhibition abilities, and rule learning. The authors explored the relationship between complexity, sympathovagal balance, and age in regulating impulsive reactions during cognitive tests, suggesting future applications of this methodology in assessing age-related cognitive decline and the brain–heart interaction.

In “Sample, Fuzzy, and Distribution Entropies of Heart Rate Variability: What Do They Tell Us about Cardiovascular Complexity?” [3], the authors compared three entropy metrics, often used in the HRV literature without properly considering their respective characteristics. The three metrics—Sample, Fuzzy, and Distribution Entropy—were applied to quantify the degree of unpredictability of heart rate series recorded in able-bodied and spinal cord-injured participants, separately in two postures. The authors compared entropy changes associated with the expected variations in HRV unpredictability that follow the postural shift, and with the expected alterations in cardiovascular complexity induced by the spinal lesion. The article discusses the robustness, sensitivity, and clinical interpretability of these entropy measures, emphasizing that, taken together, they complement each other.

An application of complex network analysis is presented in “Visibility Graph Analysis of Heartbeat Time Series” [4]. The authors applied a graph-based approach to HRV analysis. This method associates a complex network to the heart rate series, where a node of the network corresponds to a value of the series. The degree of connectivity among nodes may reveal subtle properties of the time-series dynamics, and the authors showed that the visibility graphs discriminate between young and old participants, between active and sedentary individuals, or between healthy subjects and heart failure patients.

The concept of self-organized criticality has recently been introduced to describe cardiovascular complexity. The paper “Autonomic Nervous System Influences on Cardiovascular Self-Organized Criticality” discusses how the autonomic system influences the properties of Zip’s law, a feature of self-organized criticality [5]. The authors studied beat-by-beat heart rate recordings in professional soccer players during a training session, revealing an association between Zip’s law characteristics and autonomic adjustments.

Finally, the study “Entropy-Based Multifractal Testing of Heart Rate Variability during Cognitive-Autonomic Interplay” applies a multifractality analysis to HRV recordings of volunteers undertaking three cognitive tasks (Stroop color and word task, stop-signal, and go/no-go) [6]. This contribution discusses the implications of cardiac complexity changes in relation to the interaction between the cognitive tasks and autonomic modulation.

On the central nervous system side, five studies use the electroencephalogram (EEG) as a window into brain complexity. The study “Spatio-Temporal Fractal Dimension Analysis from Resting State EEG Signals in Parkinson’s Disease” performed a four-dimensional fractal analysis of the EEG tracings in Parkinson’s disease patients and healthy controls [7]. This work provides evidence that such a complexity approach may characterize the specific changes in brain dynamics associated with Parkinson’s disease.

In “A Resource-Efficient Multi-Entropy Fusion Method and Its Application for EEG-Based Emotion Recognition”, the authors propose an entropy-based method for classifying emotional states with low computational costs [8]. Such an approach is promising for portable applications in mental health monitoring, human–computer interaction, and affective computing. The study “Improved EEG-Based Emotion Classification via Stockwell Entropy and CSP Integration” also uses complex EEG analysis for emotion recognition [9]. This work analyzed a publicly available dataset of EEG traces in volunteers while they watched videos encompassing positive, neutral, and negative emotions to induce coherent emotional responses. The authors demonstrate that the integration between Stockwell entropy and spatial filtering allows for obtaining a satisfactory affective classification.

In “Dynamic Evolution of EEG Complexity in Schizophrenia Across Cognitive Tasks”, the authors employed Higuchi Fractal Dimension, a measure of signal complexity, to examine the temporal dynamics of EEG activity across five cortical regions during an attentional and a memory-based task in individuals diagnosed with schizophrenia and healthy controls [10]. They found that, while a consistent pattern of higher neural complexity characterizes the attentional task across the different brain regions in controls, by contrast, the EEG complexity varies in a task-dependent manner in schizophrenic patients, highlighting the need for dynamic rather than static approaches.

The study “Brain Complexity and Parametrization of Power Spectral Density in Children with Specific Language Impairment” compared measures of EEG complexity in children diagnosed with specific language impairment and in normo-developed children of the same age [11]. The results suggested alterations associated with the cognitive and linguistic characteristics of this disease, in the excitatory–inhibitory balance and intra- and inter-hemispheric connectivity.

Alongside EEG, the interactions between cerebral hemodynamics and respiration were explored in “Time-Resolved Information-Theoretic and Spectral Analysis of fNIRS Signals from Multi-Channel Prototypal Device” [12]. The authors applied informational and spectral measures to multichannel fNIRS signals during a breath-holding task to characterize the impact of respiratory activity on scalp hemodynamics within the framework of Network Physiology. They highlighted distinct informational dynamics across the breathing and apnea phases, opening new perspectives on the use of complexity for optical neuroimaging.

Another complex physiological system considered in this Special Issue concerns motor control and movement. The study “Multifractal Multiscale Analysis of Human Movements during Cognitive Tasks” explored the regularity of cycling movements by multiscale multifractality during progressive cognitive loads [13]. This study revealed how cognitive load alters the complexity of motor control, suggesting new perspectives for assessing coordination structures between higher neural centers and movement.

Finally, at the theoretical level, “A Spike Train Production Mechanism Based on Intermittency Dynamics” introduces a generative spike-train model of the neuron that reproduces high-frequency spontaneous membrane potential fluctuations by coupling intermittent maps, which are nonlinear first-order difference equations [14]. Such a model may prove to be useful as a test bed for complexity metrics in neurology.

Overall, this Special Issue presents a variety of applications and methodologies: from the heart to the brain, from electrical to hemodynamic and motion signals, and from entropic to multifractal measures. Emerging challenges include the need for robust metrics for short and noisy series, the standardization of the procedures, and the physiological interpretability of results, a prerequisite for clinical translation. The contributions collected here, however, demonstrate that complexity is not just an abstract concept but an attribute of living systems, which, if correctly described and quantified, might allow a better understanding of the physiological processes, possibly becoming a practical tool for aiding clinical interventions.
